# Biochanin A Inhibits the Growth and Biofilm of *Candida* Species

**DOI:** 10.3390/ph17010089

**Published:** 2024-01-09

**Authors:** Monika Janeczko, Elżbieta Kochanowicz

**Affiliations:** Department of Molecular Biology, Faculty of Medicine, The John Paul II Catholic University of Lublin, Konstantynów 1i, 20-708 Lublin, Poland; mazure@kul.pl

**Keywords:** biochanin A, biofilm, *Candida*, hyphae, qRT-PCR, cell membrane integrity, cell wall damage, flavonoids

## Abstract

The aim of this study was to investigate the antifungal activity of biochanin A (BCA) against planktonic growth and biofilms of six *Candida* species, including *C. albicans*, *C. parapsilosis*, *C. glabrata*, *C. tropicalis*, *C. auris*, and *C. krusei*. We applied various assays that determined (a) the antimicrobial effect on growth of *Candida* species, (b) the effect on formation of hyphae and biofilm, (c) the effect on the expression of genes related to hyphal growth and biofilm formation, (d) the influence on cell wall structure, and (e) the effect on cell membrane integrity and permeability. Moreover, disk diffusion tests were used to investigate the effect of a combination of BCA with fluconazole to assess their possible synergistic effect on drug-resistant *C. albicans*, *C. glabrata*, and *C. auris.* Our results showed that the BCA MIC_50_ values against *Candida* species ranged between 125 µg/mL and 500 µg/mL, and the MIC_90_ values were in a concentration range from 250 µg/mL to 1000 µg/mL. The treatment with BCA inhibited adhesion of cells, cell surface hydrophobicity (CSH), and biofilm formation and reduced hyphal growth in all the analyzed *Candida* species. Real-time qRT-PCR revealed that BCA down-regulated the expression of biofilm-specific genes in *C. albicans*. Furthermore, physical destruction of *C. albicans* cell membranes and cell walls as a result of the treatment with BCA was observed. The combination of BCA and fluconazole did not exert synergistic effects against fluconazole-resistant *Candida*.

## 1. Introduction

Biochanin A (5,7-dihydroxy-4′-methoxy-isoflavone, BCA) is a flavonoid mainly found in red clover, cabbage, lucerne, chickpea, peanuts, legumes, and many other herbal products [1]. BCA is the methylated precursor of the isoflavone genistein, which is another well-studied compound. In the gut, intestinal bacteria convert BCA to its demethylated form [2]. However, the biological effects of BCA observed in vitro and in vivo are not identical to those of genistein. BCA is known to have various effects and biological activities, including anti-inflammatory, antioxidant, anti-infective, and anti-cancer properties, glucose and lipid metabolism modulatory activity, and cancer preventive, neuroprotective, and drug interaction effects [1]. BCA plays complex roles in the regulation of multiple biological functions by binding DNA and some specific proteins or acting as a competitive substrate for some enzymes [3,4,5,6]. In addition, BCA is used in treatment of estrogen deficiency and pain and to reduce the severity of nerve damage [7,8]. Interestingly, plant extracts containing BCA are used as dietary supplements, especially in alleviating postmenopausal symptoms in women, which is related to the fact that isoflavones are known to act as phytoestrogens because of their close resemblance to estrogen [4,9].

Among its antimicrobial properties, BCA was found to have antiviral potential and selective antibacterial and anti-parasitic activities. It inhibited the replication of the H5N1 virus and blocked the H5N1-induced activation of ERK1/2, Akt, and NF-κB and the virus-induced production of IL-6/-8/-10 [10]. BCA has been reported to possess antibacterial activity against *C. pneumoniae*, *Clostridia* spp., and intracellular bacteria belonging to the genus *Chlamydia* spp. [11,12]. Furthermore, BCA has been shown to induce AMPK/ULK1/mTOR-mediated autophagy and macrophage extracellular traps, which enhance the defense against *Salmonella* sp. infection [13]. A recent study has demonstrated the antibacterial properties of BCA against *Staphylococcus aureus* [14]. Moreover, BCA has been reported to exert a synergistic effect with ciprofloxacin on the efflux system of resistant strains of *S. aureus* [15]. Synergistic antimicrobial activity of BCA with fluoroquinolones against antibiotic-resistant *Ureaplasma* spp. has been described as well [16]. BCA has been investigated as a mycobacterial efflux pump inhibitor in *Mycobacterium smegmatis* [17]. In addition, it has been reported to possess antileishmanial properties against promastigotes of *Leishmania chagasi* [18]. However, its antifungal activity, especially against *Candida* species, has been studied to a limited extent, which emphasizes the importance of this work [19,20,21,22].

*Candida* species are the causative agents of most fungal infections in humans. These species are represented by *Candida albicans*, the most common cause of opportunistic infections, drug-resistant *Candida glabrata*, *Candida auris*, which is a new global threat to public health, and other emerging species such as *Candida tropicalis*, *Candida parapsilosis*, and *Candida krusei* [23,24]. The importance of these species for global public health was confirmed by the World Health Organization (WHO). On 25 October 2022, the WHO released the first edition of fungal priority pathogens list, and *Candida* species were listed in the Critical Priority Group (*C. albicans* and *C. auris*), High Priority Group (*C. glabrata*, *C. tropicalis*, and *C. parapsilosis*), and Medium Priority Group (*C. krusei*) [25].

*Candida* species are commensal yeasts present in a substantial part of the human population, asymptomatically colonizing the skin and oral, gastrointestinal, and reproductive tracts [26]. However, upon disruption of the barrier integrity and host immune response, the fungus can migrate through the epithelium and access deep tissues to cause infection [24,27]. In medical terms, the most important infections caused by *Candida* are mucosal and systematic infections. Mucocutaneous surfaces that are primarily affected by these species are the vagina (vulvovaginal candidiasis), oral cavity (oropharyngeal candidiasis), esophagus (esophageal candidiasis) and, less commonly, nails (onychomycosis) [27]. Systemic candidiasis may involve the internal organs, blood circulatory system, and central nervous system. It may also affect the abdominal cavity with or without dissemination to the bloodstream [24]. Diabetic and cancer patients, as well as severely ill patients with a protracted hospital stay, frequent use of intravascular catheters, prolonged antibiotic therapy, and immunocompromised status, are especially susceptible to *Candida* infection [28,29,30].

*Candida* species employ several virulence traits to effectively cause disease and withstand the subsequent antifungal host response and antifungal drug treatment [27]. The most important virulence and adaptive factors are (a) plasticity in switching between different morphogenetic states; (b) adhesion, invasion, and cell damage; (c) biofilm formation; (d) temperature and pH adaptation; (e) nutrient acquisition; (f) immune evasion; and (g) drug tolerance [27,31]. The majority of manifestations of candidiasis are related to biofilm formation on the surface biological or artificial surfaces. The US National Institute of Health estimates that approximately 80% of microbial infections are associated with a biofilm etiology, including *C. albicans* infections [31,32]. Biofilms are highly organized attached microbial communities surrounded by a matrix containing exopolymeric substances (carbohydrates, proteins, lipids, and DNA). The *Candida* biofilm development can be divided into four major phases: (a) adherence, during which yeast cells attach to a material surface and form a basal layer of the biofilm; (b) proliferation, which is characterized by the initiation of filamentation, leading to the emergence of hyphal and pseudo-hyphal cells; (c) maturation, when the hyphal scaffold become encased in a layer of self-produced exopolymeric substances; and (d) dispersal, involving the release of yeast cells that seed new sites of infection [33]. The complexity of the biofilm structure facilitates the influx of nutrients, the removal of waste products, and the formation of microniches throughout the biofilm [31,34]. The main clinical consequences of biofilm formation that have an adverse effect on the management of patients with these infections are the increased resistance of the cells in the biofilm to antifungal treatment and their protection against host defenses [33,34].

The currently available classes of antifungals for the treatment of *Candida* infection include polyenes, azoles, echinocandins, pyrimidines, and allylamines. These substances act on different fungal cell targets: polyenes bind ergosterol, altering the permeability of the cell membrane, azoles target lanosterol 14α-demethylase enzymatic activity, thus decreasing ergosterol content in the cell membrane, echinocandins target 1,3-*β*-glucan synthase activity, thus damaging the cell wall, pyrimidines disrupt DNA and RNA biosynthesis by interfering with pyrimidine metabolism, and allylamines act by attenuating an enzyme (squalene epoxidase) of the ergosterol synthesis pathway [30,35]. Although the substances from these groups are currently used effectively in the treatment of *Candida* infections, there are some disadvantages to their use. Overuse, prolonged treatment, and environmental exposure to azoles, polyenes, and echinocandins have recently led to the emergence and spread of drug resistance. In particular, the incidence of *Candida* species resistance to azoles and echinocandins is increasing [35]. *C. albicans* cells, especially within a biofilm, display high levels of resistance to azoles and polyenes. The major contributors to resistance in the biofilm are the extracellular matrix, increased cell density, overexpression of efflux pumps, changes in the sterol composition of the cell membrane, and presence of a subpopulation of persister cells that can tolerate high concentrations of antifungals [31,36]. Several of the non-*C. albicans Candida* species, such as *C. krusei*, are intrinsically resistant or less susceptible to several classes of antifungals, whereas others, including *C. glabrata*, develop acquired resistance following exposure to antifungal agents. Resistance to more than one drug class (multidrug resistance) remains uncommon but has been increasingly reported, e.g., in *C. auris* [37]. According to the report generated in 2019 by the Centers for Disease Control and Prevention (CDC), there were 34,800 cases of infection and 1700 deaths caused by drug-resistant *Candida* spp. In the United States [35,38].

The large number of fungal Infections, the mortality associated with invasive fungal infections, and the shortcomings of currently used antifungal drugs have prompted a growing need to discover new, more effective drugs with minimal or manageable toxicities/side effects [35]. Recently, natural compounds have been an important choice for the development of antifungal and anti-biofilm agents due to their low toxicity and diverse biological properties. Flavonoids, i.e., a major class of natural compounds known as polyphenols, are secondary metabolites naturally occurring in plants [39,40]. Epidemiological and clinical studies have reported the majority of polyphenols to exhibit antimicrobial activities [14,41]. Several studies have demonstrated the potential of flavonoids (e.g., quercetin, myricetin, kaempferol, rutin, baicalein, licochalcone A, and genistein) to control *C. albicans* growth and biofilm formation [42,43,44,45,46,47].

The aim of this study was to explore the anti-hyphal and anti-biofilm activity of BCA against *Candida* species with reference to the inhibition of cell adhesion and biofilm formation and eradication of established biofilms. In addition, qRT-PCR analysis of *C. albicans* cells was performed to provide a molecular explanation of BC’’s potential for biofilm inhibition. Additionally, the influence of BCA on the yeast cell wall and cytoplasmic membrane was examined as one of the possible mechanisms of its antifungal activity. Moreover, the synergy testing method was used to establish of effects of BCA and fluconazole used in combination.

## 2. Results

### 2.1. Antifungal Susceptibility of Planktonic Cells

We investigated the effect of BCA on the growth of six *Candida* species: *C. albicans*, *C. parapsilosis*, *C. glabrata*, *C. tropicalis*, *C. auris*, and *C. krusei.* The antifungal activity of BCA is presented in Table 1. The MIC_50_ endpoints ranged from 125 to 500 µg/mL and the MIC_90_ values ranged from 250 to 1000 µg/mL. *C. krusei* was found to be most susceptible to BCA, and *C. glabrata* was the least susceptible to the tested compound.

### 2.2. Effect of BCA on Adhesion of Cells

**Figure 1:**
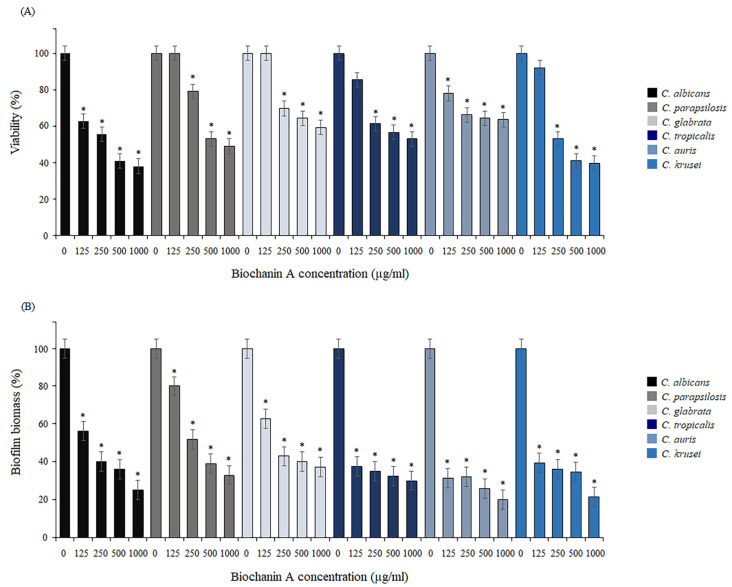
Inhibition of the adherence phase. Biofilm reduction relative to the control untreated *Candida* cells quantified by the MTT assay (**A**) and crystal violet staining (**B**). Data are expressed as mean ± standard error of three independent experiments. * *p* < 0.05 significance compared to the control (untreated cells) set to 100%.

The effect of BCA on the adhesion of *Candida* cells was evaluated by MTT assay and crystal violet staining. Adherence was initiated in 96-well microtiter plates in the presence of serially double-diluted concentrations of BCA, and the plates were incubated at 37 °C for 2 h. The viability and biomass of cells attached in the adherence phase were expressed as a percentage in relation to the controls (untreated cells). The data from the MTT reduction assay showed that BCA decreased the viability of all *Candida* species during the adherence phase in a dose-dependent manner. In the case of *C. albicans*, under the influence of BCA at concentrations ranging from 125 µg/mL to 1000 µg/mL, there was a reduction in the viability of cells forming the biofilm to 62.8–38.1%. No significant differences were observed in the growth of *C. parapsilosis* and *C. glabrata* treated with BCA at 125 µg/mL, compared to the untreated yeast cells. The higher concentrations of BCA (250–1000 µg/mL) caused a reduction in cell viability in the ranges of 49–79% and 59.3–69.7% in *C. parapsilosis* and *C. glabrata*, respectively. The populations of living cells of *C. tropicalis*, *C. auris*, and *C. krusei* under the influence of BCA used at concentrations of 125–1000 µg/mL were reduced to 53–85.5%, 63.6–78%, and 39.7–92%, respectively (Figure 1A). Based on the crystal violet assay, we found that BCA at concentrations of 125–1000 µg/mL reduced the adherent cell biomass of C. *albicans*, *C. parapsilosis*, and *C. glabrata* to 25–56%, 32.9–80%, and 37–62.8%, respectively. The best results were obtained in the case of *C. tropicalis*, *C. auris*, and *C. krusei*, which were inhibited by BCA (125–1000 µg/mL) in the adherence phase up to 30–37.5%, 20–31.4%, and 2.5–39.2%, respectively (Figure 1B).

**Figure 2:**
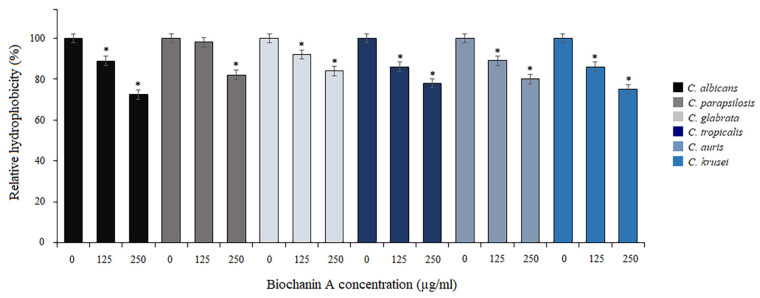
Relative cell surface hydrophobicity (CSH) when biochanin A was used at 125 µg/mL and 250 µg/mL against *Candida* species. Data are expressed as mean ± standard error of three independent experiments. * *p* < 0.05 significance compared to the control (untreated cells) set to 100%.

Knowing that there is a correlation between cell surface hydrophobicity (CSH) and adhesion of *Candida*, we further explored the effect of BCA on the hydrophobic properties of fungal cells. The influence on CSH was measured as the percentage of cell adsorption to octane and presented as relative hydrophobicity (Figure 2). Compared to the controls (untreated cells), BCA at 125 µg/mL reduced CSH to 86–98%, depending on the *Candida* species. In turn, the higher concentrations of BCA (250 µg/mL) reduced CSH to 72.4–84%, depending on the *Candida* species (Figure 2).

### 2.3. Activity of BCA against Biofilm Formation and Mature Biofilm

**Figure 3:**
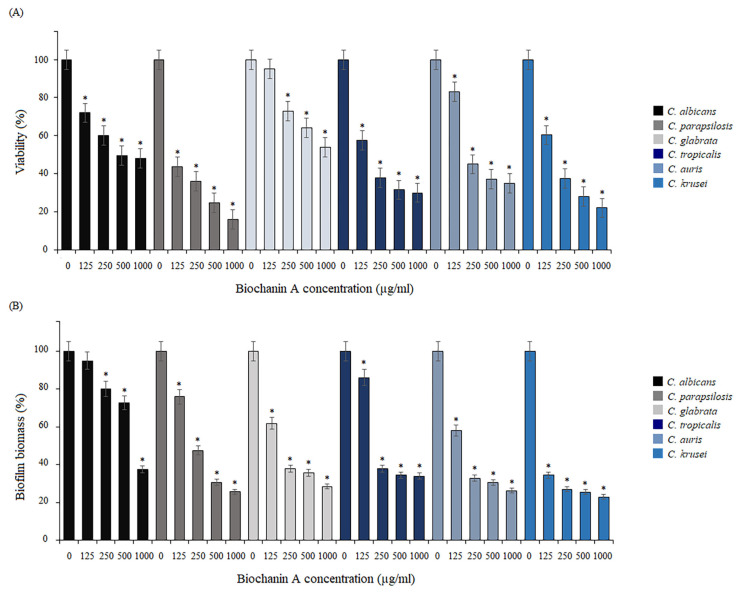
Effect of biochanin A on biofilm formation. Biofilm reduction relative to the control untreated *Candida* cells quantified by the MTT assay (**A**) and crystal violet staining (**B**). Data are expressed as mean ± standard error of three independent experiments. * *p* < 0.05 significance compared to the control (untreated cells) set to 100%.

The activity of BCA was tested in terms of the formation of biofilm by reference *Candida* species. Biofilms were formed in the presence of serially double-diluted concentrations of BCA for 24 h. The colorimetric assays of the cell biomass of all species showed that BCA inhibited biofilm formation in a dose-dependent manner (Figure 3A,B). In the case of *C. albicans*, under the influence of BCA at concentrations of 125–1000 µg/mL, the viability of biofilm cells was reduced to 48–72% and the biofilm biomass decreased to 37.5–95%. In turn, the viability of the *C. parapsilosis* biofilm was reduced to 16–43.6%. Based on the CV staining, the reduction in the biofilm biomass of this species was up to 25.7–76%, compared to the control (untreated cells). After the incubation of *C. glabrata* biofilm cells with BCA in the tested concentration range, the biofilm was reduced to 53.8–95% in the MTT test and to 28.5–61.9% in the CV assay. As shown by our results of the MTT test, the *C. tropicalis* biofilm growing under the BCA pressure was reduced to 30–57.5%. In turn, the CV staining revealed that the biofilm biomass was reduced to 34–86.2%. In the tested concentration range (125–1000 µg/mL), BCA also showed activity against biofilm formation by *C. auris.* It was found that BCA inhibited the viability and biomass of biofilms of this species with similar intensity as in the other *Candida* species. The MTT assay revealed that the number of viable cells in the biofilms decreased to 35–83%. As shown by the staining of the biofilm cells with crystal violet, BCA reduced of biofilm biomass to 26.3–58% in a dose-dependent manner. In the case of *C. krusei*, under the influence of BCA applied at 125–1000 µg/mL, the viability of biofilms was reduced to 22–60.3%, and the biomass of the biofilms decreased to 23–34.5%.

**Figure 4:**
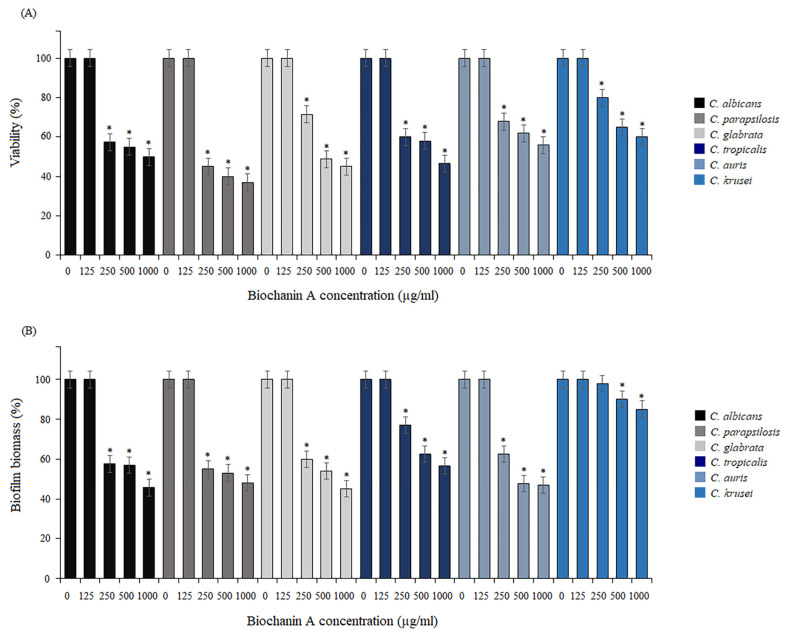
Eradication of mature biofilms. Biofilm reduction relative to the control untreated *Candida* cells quantified by the MTT assay (**A**) and crystal violet staining (**B**). Data are expressed as mean ± standard error of three independent experiments. * *p* < 0.05 significance compared to the control (untreated cells) set to 100%.

To assess the antifungal effect of BCA against the mature biofilms formed by the reference strains, susceptibility tests against the preformed biofilms were carried out. Biofilms were allowed to form for 24 h at 37 °C. Next, serially double-diluted concentrations of BCA were added to these biofilms and the samples were incubated at 37 °C for 24 h. As shown by the reactions with MTT and the staining of the biofilm cells with crystal violet, BCA significantly induced destabilization of the mature biofilms (Figure 4A,B). Based on both assays, BCA at the concentration of 125 µg/mL showed no inhibitory activity against mature biofilm formed by all tested *Candida* species. The cell viability of the mature *C. albicans* biofilm treated with BCA at concentrations of 250–1000 µg/mL was reduced to 50–57.5% compared to the untreated cells (control). Similar results were achieved for *C. glabrata*, *C. tropicalis*, and *C. auris*, where in the same BCA concentration ranges (250–1000 µg/mL) there was a reduction in viability in the range of 45–71.5%, 46.6–60%, and 56–68%, respectively. The anti-biofilm activity of BCA against the biofilm formed by *C. parapsilosis* was the strongest, leading to an up to 37–45% reduction in cell viability compared to the control, while the weakest BCA activity was noted in the biofilm formed by *C. krusei*, where a 58–80% reduction in cell viability was achieved (Figure 4A). The CV staining tests confirmed these results, as the mature biofilm biomass under the pressure of BCA at 250–1000 µg/mL was reduced by 45.7–57.6%, 48–55%, 45–60%, 56.7–77%, 47–62.5%, and 85–98% in *C. albicans*, *C. parapsilosis*, *C. glabrata*, *C. tropicalis*, *C auris*, and *C. krusei*, respectively (Figure 4B).

### 2.4. Inhibitory Activity of BCA against the Transition of Candida from Yeast to Hyphae

**Figure 5:**
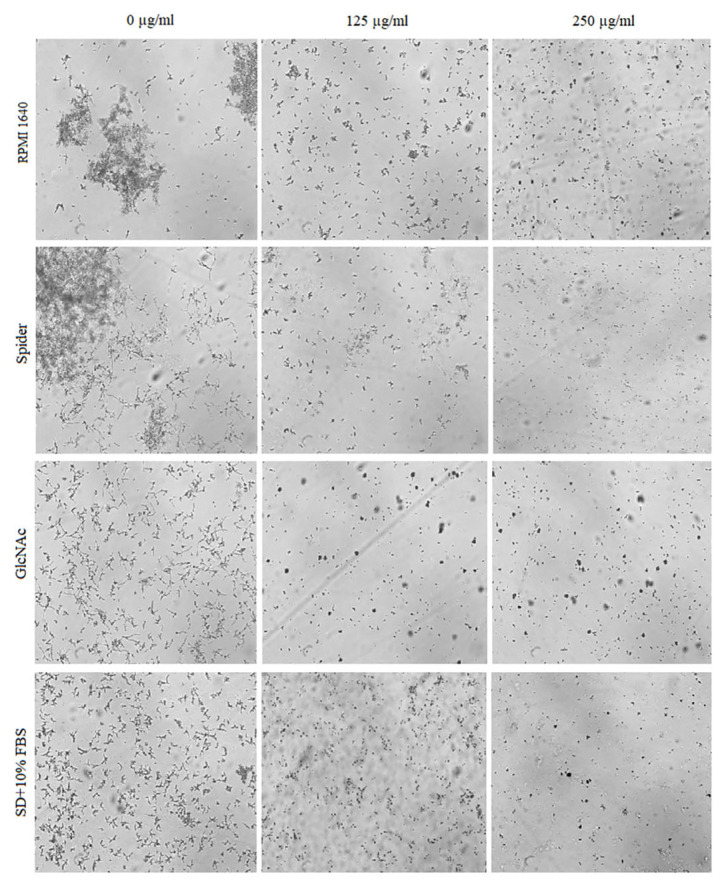
Cellular morphologies of *C. albicans* in different hyphal-inducing media under BCA pressure at concentrations of 125 µg/mL and 250 µg/mL.

**Figure 6:**
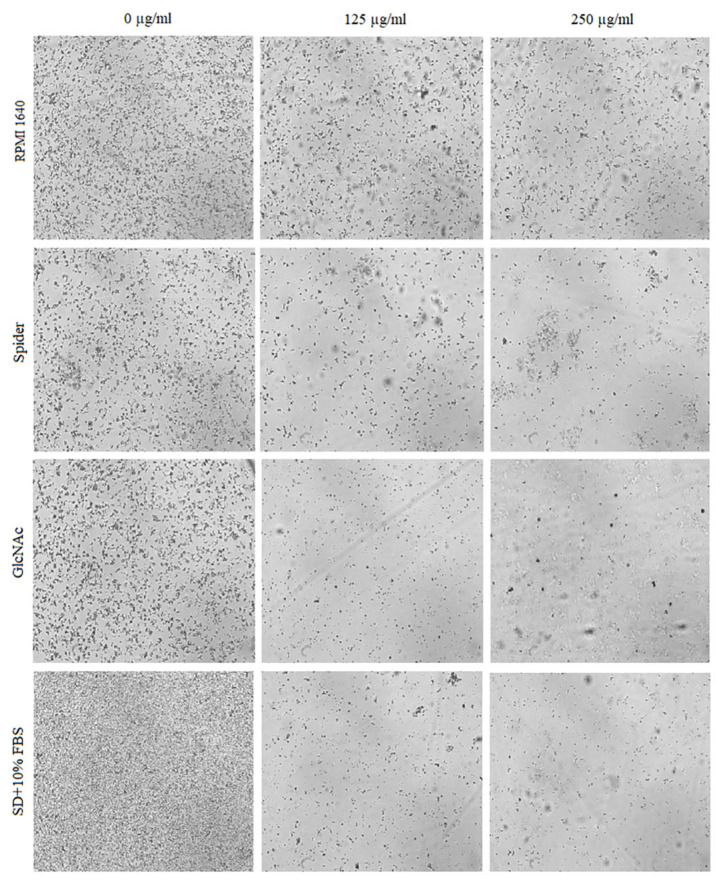
Influence of different concentrations of BCA (125 µg/mL and 250 µg/mL) on hyphal formation by *C. parapsilosis* in different hyphal-inducing media.

**Figure 7:**
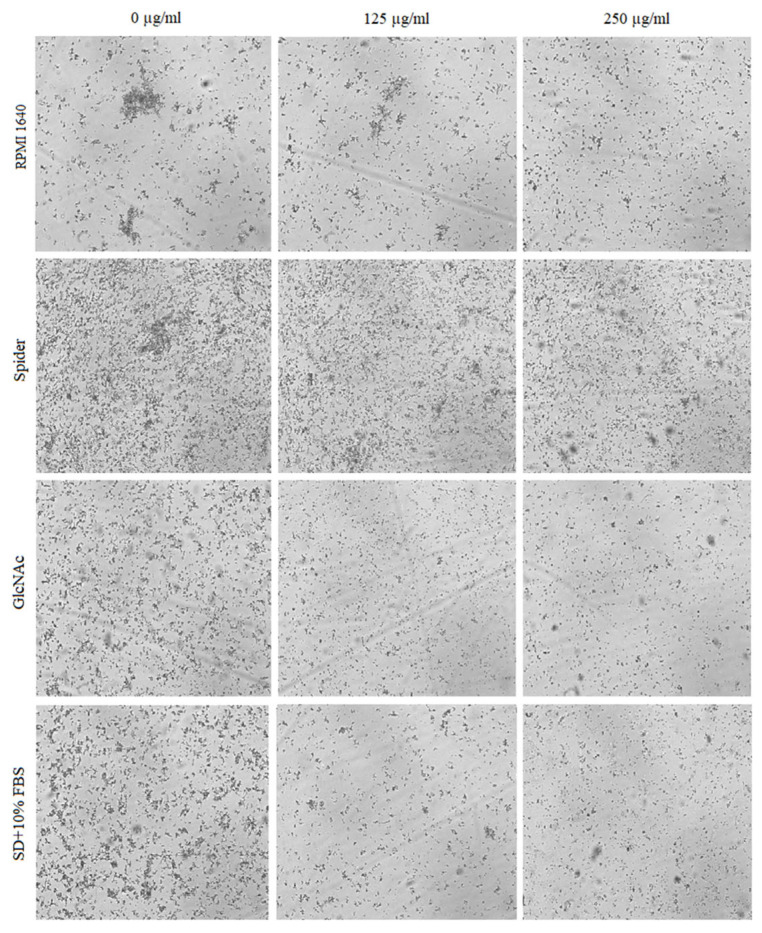
Pseudohyphal formation of *C. tropicalis* inhibited by BCA at concentrations of 125 µg/mL and 250 µg/mL in different hyphal-inducing media.

**Figure 8:**
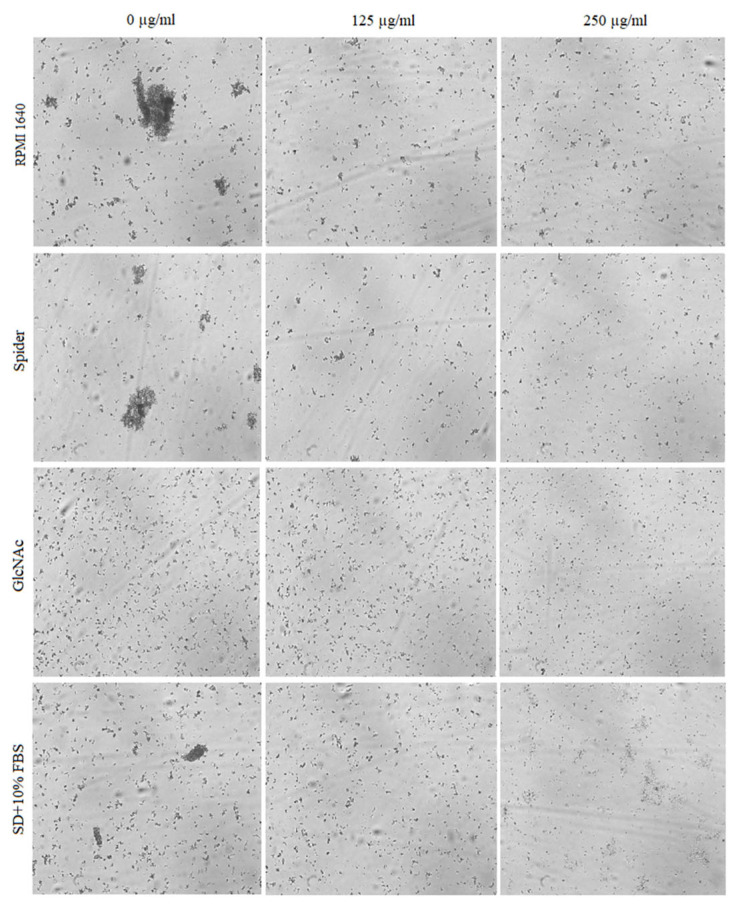
Cellular morphologies of *C. auris* in different hyphal-inducing media under BCA pressure at concentrations of 125 µg/mL and 250 µg/mL.

**Figure 9:**
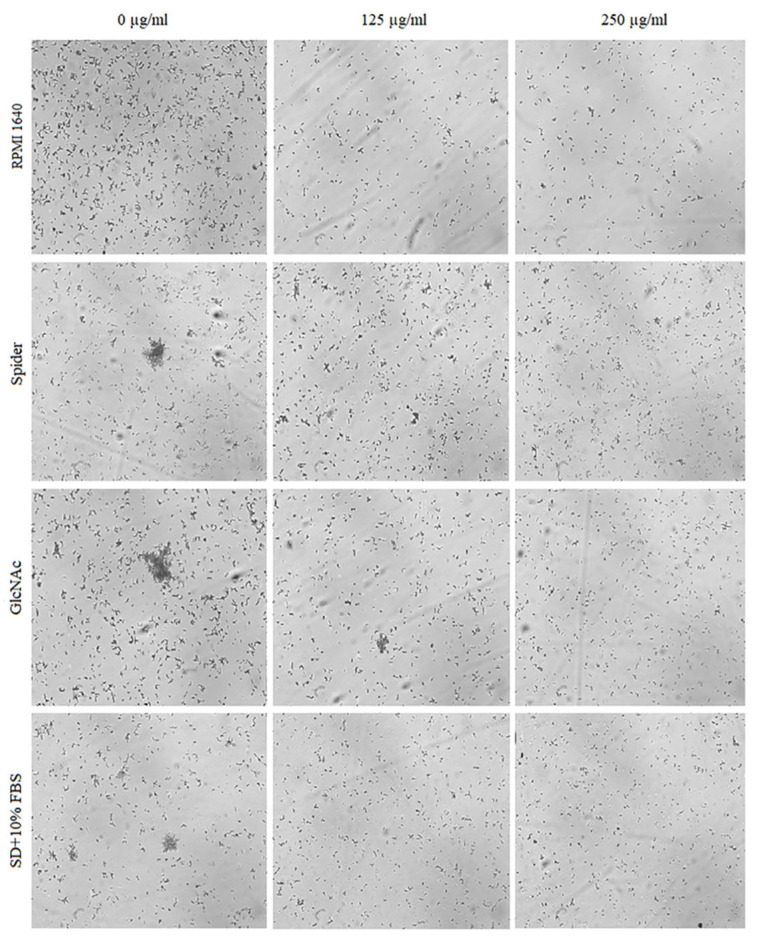
Influence of different concentrations of BCA (125 µg/mL and 250 µg/mL) on hyphal formation by *C. krusei* in different hyphal-inducing media.

The effect of BCA on the hyphal and pseudohyphal growth in five *Candida* species (*C. albicans*, *C. parapsilosis*, *C. tropicalis*, *C. auris*, and *C. krusei*) was studied at concentrations of 125 µg/mL and 250 µg/mL in liquid hyphal-inducing media, i.e., RPMI 1640 medium, Spider medium, GlcNAc medium, and SD medium with 10% FBS. After 4 h of incubation at 37 °C, the cellular morphologies of *Candida* species were photographed using an inverted microscope. Our results indicated that each of the tested *Candida* species was characterized by a different tendency to filamentous growth in the different media (Figure 5, Figure 6, Figure 7, Figure 8 and Figure 9).

The yeast-to-hyphal transition was considered as a crucial virulence factor of *C. albicans*. *C. albicans* yeasts are truly polymorphic due to their ability to form hyphae and/or pseudohyphae [48]. As shown in Figure 5, BCA reduced the hyphae in all the four hyphal-inducing media. BCA inhibited hyphal growth in GlcNAc medium and in FBS-containing SD medium at the concentration of 125 µg/mL, and completely blocked hyphal growth in all the media at the concentration of 250 µg/mL.

*C. parapsilosis* does not produce true hyphae, but can generate pseudohyphae that are characteristically large and curved and often referred to as “giant cells” [49]. As shown in Figure 6, *C. parapsilosis* exhibited lower hyphal growth intensity in the control samples (untreated cells) compared to *C. albicans*. Under the influence of BCA, reduction of hyphal cells was noted in all the media except RPMI 1640 medium.

*C. tropicalis* produces oval blastospores, pseudohyphae depending on some reports, and true hyphae [49,50]. As shown in Figure 7, the *C. tropicalis* cells in the control probes in all the hyphal-inducing media showed intensive hyphal growth. A significant reduction of hyphae under the influence of BCA applied at concentrations of 125 µg/mL and 250 µg/mL was observed only in GlcNAc medium and SD medium with 10% FBS. In the case of the growth of this species in RPMI 1640 medium and Spider medium, no inhibition of hyphal formation was observed.

*C. auris* does not form hyphae and pseudohyphae, but under certain special cultivation conditions, it is able to grow into a pseudohyphae-like form [50]. The growth of *C. auris* is presented in Figure 8. Intensive pseudohyphal formation and strong aggregation of cells were especially noticeable in RPMI 1640 medium, Spider medium, and SD plus 10% FBS medium (controls). In the case of GlcNAc, no aggregation of cells was observed. In all samples treated with BCA at concentrations of 125 µg/mL and 250 µg/mL, a significant reduction in the number of cells and reduction of pseudohyphae form were visible.

*C. krusei* is able to create hyphae and pseudohyphae [49,50]. We also observed a large number of *C. krusei* hyphae in the control groups (untreated cells) in all the hyphal-inducing media. Additionally, the incubation of *C. krusei* in Spider medium, GlcNAc medium, and SD with 10% FBS medium clearly resulted in the formation of hyphal cell agglomerates. As expected, we confirmed the significant inhibition of the hyphal formation when *C. krusei* was incubated in the presence of BCA at concentrations of 125 µg/mL and 250 µg/mL compared to the control groups (Figure 9).

In contrast to other *Candida*, *C. glabrata* is not polymorphic and grows only as blastoconidia (yeast) [50]. Hence, the study of morphological changes related to the inhibition of hyphal and pseudohyphal growth by BCA in this *Candida* species was unjustified.

### 2.5. Effect of BCA on C. albicans Gene Expression

**Figure 10:**
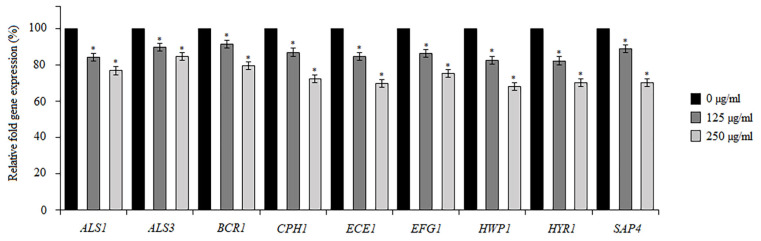
Changes in the gene expression of some important biofilm formation-related genes. The level of gene expression is displayed after normalization with the internal control housekeeping gene *ACT1*. Data are expressed as mean ± standard error of three independent experiments. * *p* < 0.05 significance compared to the control (untreated cells) set to 100%.

To understand the anti-biofilm mechanism of BCA, we further investigated changes in the expression of known adhesion-related, hypha-related, and biofilm-related genes after the BCA treatment using real-time qRT-PCR. In Spider medium at 37 °C, hypha-specific and adhesion-specific genes *ALS1*, *ALS3*, *ECE1*, *HWP1*, *HYR1*, and *SAP4* were down-regulated after the BCA treatment. More precisely, BCA used at 125 µg/mL reduced the expression of these genes to 84.6%, 90%, 84.8%, 82.9%, 82.5%, and 89.1%, respectively. In turn, BCA applied at 250 µg/mL lowered the expression of these genes to 77%, 85%, 69.9%, 68.4%, 70%, and 70.1%, respectively. The levels of two master transcriptional regulators, i.e., the *BCR1* and *EFG1* genes, decreased after the treatment with BCA at 125 µg/mL and 250 µg/mL to 91.6–79.7% and 86.5–75.5%, respectively. Another regulation gene, namely *CPH1*, was also down-regulated 1.16- and 1.38-fold compared to the control (Figure 10). Taken together, the real-time qRT-PCR results indicated that the BCA treatment down-regulated the expression of some adhesion-related and biofilm-specific genes and some genes known to regulate the yeast-to-hypha transition.

### 2.6. Effect of Biochanin A on the Cell Wall and Cell Membrane of C. Albicans

**Figure 11:**
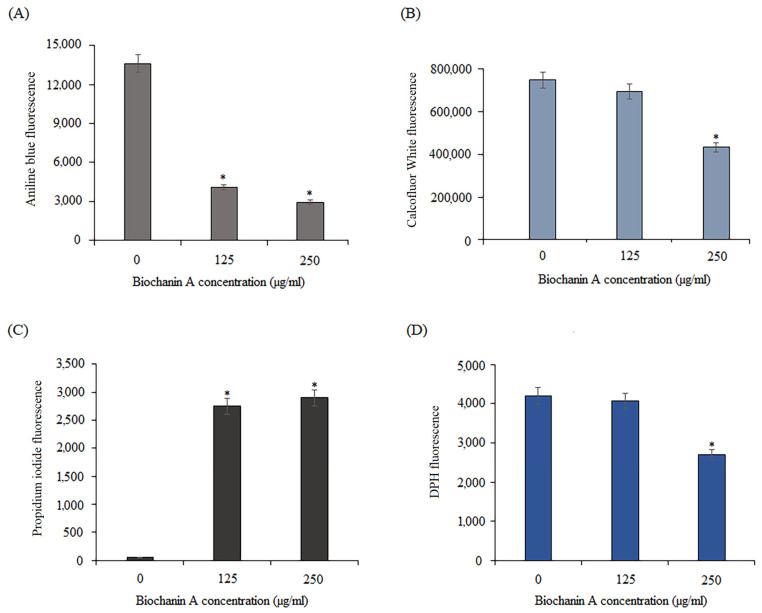
Effect of BCA on the *C. albicans* cell wall and cell membrane. (**A**) Loss of aniline blue fluorescence in the cell wall (λ_Ex_ = 370 nm/λ_Em_ = 509 nm). (**B**) Changes in calcofluor white fluorescence in the cell wall (λ_Ex_ = 370 nm/λ_Em_ = 440 nm). (**C**) Uptake of propidium iodide by cells (λ_Ex_ = 488 nm/λ_Em_ = 617 nm). (**D)** Influence on the ability of DPH to bind to the hydrophobic core of the membrane (λ_Ex_ = 360 nm/λ_Em_ = 460 nm). Data are expressed as mean ± standard error of three independent experiments. * *p* < 0.05 significance compared to the controls (untreated cells).

The influence of BCA on the cell wall of *C. albicans* was examined using aniline blue and calcofluor white fluorescence probes. Aniline blue binds to (1,3)-*β*-D-glucans in the cell wall, while calcofluor white interacts with chitin. The yeasts were grown in the presence of BCA at concentrations of 125 µg/mL and 250 µg/mL. The control contained DMSO at the same concentrations as the samples with BCA. As shown in Figure 11A, the intensity of fluorescence of fungal cells stained with blue aniline was lower in the BCA-treated cells than in the controls, which indicates the loss of glucans in the cell wall. Moreover, as shown in Figure 11B, a significant loss of fungal cell wall fluorescence was also visible after the treatment with BCA at the concentration of 250 μg/mL stained with calcofluor white. In turn, the treatment of *C. albicans* cells with BCA at the concentration of 125 µg/mL resulted in a virtually imperceptible reduction in the chitin content in the cell wall.

To check whether BCA also affected the permeability of the *C. albicans* cell membrane, a propidium iodide assay and a DPH assay were performed. Propidium iodide (PI), which is impermeable to cell membranes, can only enter cells with a damaged cell membrane and emits red fluorescence [51]. As shown in Figure 11C, *C. albicans* cells exposed to BCA displayed more red fluorescence than the BCA-free control. This result indicated that BCA damaged the integrity of the *Candida* cell membrane. Knowing that the enhancement of membrane permeability may be correlated with altered membrane potential, we further explored the effect of BCA on the binding of DPH to the hydrophobic core of the membranes. The binding of DPH and its derivatives to the hydrophobic core of the membranes is coupled with a strong increment in their fluorescence [52]. Therefore, the influence of BCA on the *C. albicans* plasma membrane was examined using a DPH fluorescence probe. The treatment with BCA at 125 µg/mL virtually did not change the DPH fluorescence intensity (reduction only to 95%) compared to the control (untreated cells), but BCA at 250 µg/mL resulted in a fluorescence reduction to 64.3% (Figure 11D). These results indicated that the reduction of cell wall components, changes in membrane potential, and increase in plasma membrane permeability may be potential anti-*Candida* mechanisms of BCA action.

### 2.7. Combination of BCA and Fluconazole against Fluconazole-Resistant Candida Species

**Figure 12:**
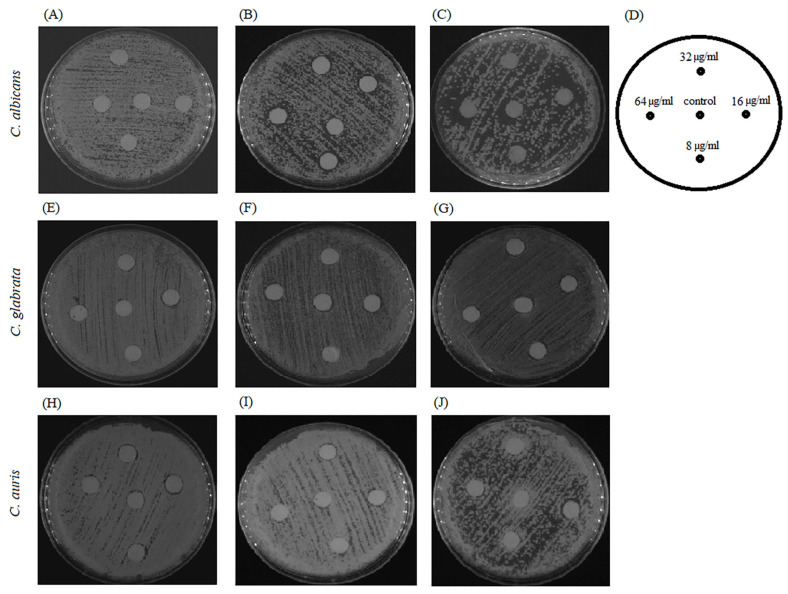
Agar disk diffusion assay of different concentrations of BCA alone or in combination with fluconazole against *C. albicans*, *C. glabrata*, and *C. auris.* Panels (**A**,**E**,**H**) show agar plates without BCA, panels (**B**,**F**,**I**) show agar plates with 125 µg/mL BCA, and panels (**C**,**G**,**J**) show agar plates containing 250 µg/mL BCA. Panel (**D**) describes the images of panels (**A**–**C**) and (**E**–**J**) containing 64 µg/mL, 32 µg/mL, 16 µg/mL, and 8 µg/mL of fluconazole or DMSO per disk as a control.

Further, we visualized the interplay of BCA and fluconazole on fluconazole-resistant strains, including *C. albicans*, *C. glabrata*, and *C. auris*, using an agar disk diffusion test. Fluconazole used at a dose of 8–64 µg per disk showed weak inhibitory activity against *Candida* species, as the halo surrounding the disk on a plain agar plate was turbid with colonies (Figure 12A,E,H). Similarly, on the agar plates containing 125 µg/mL and 250 µg/mL BCA, fluconazole did not produce clearer or larger zones at 8–64 µg per disk (Figure 12B,C,F,G,I,J).

**Figure 13:**
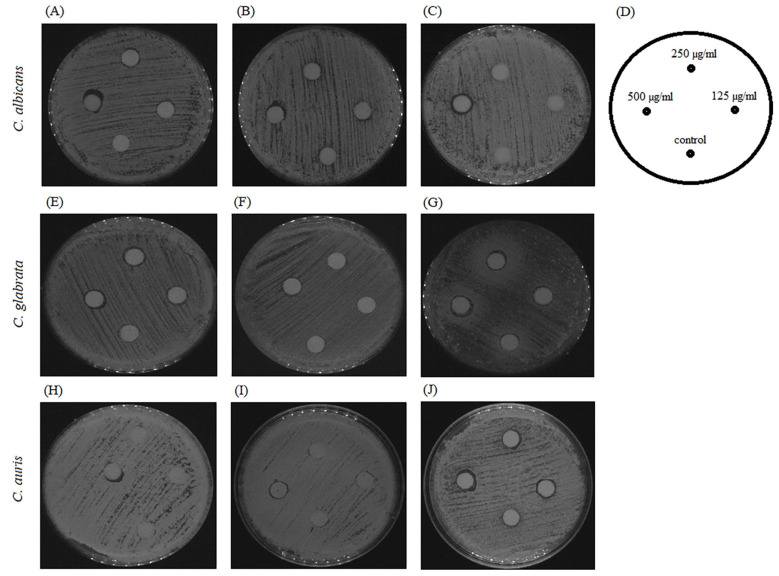
Agar disk diffusion assay of different concentrations of BCA alone or in combination with fluconazole against *C. albicans*, *C. glabrata*, and *C. auris.* Panels (**A**,**E**,**H**) show agar plates without fluconazole, panels (**B**,**F**,**I**) show agar plates with 8 µg/mL fluconazole, and panels (**C**,**G**,**J**) show agar plates containing 32 µg/mL fluconazole. Panel (**D**) describes the images of panels (**A**–**C**) and (**E**–**J**) containing 500 µg/mL, 250 µg/mL, 125 µg/mL of BCA or DMSO per disk as a control.

A similar effect was achieved when fluconazole was added to the agar medium at the sub-therapeutic concentrations of 8 µg/mL and 32 µg/mL. BCA applied at the dose of 125–500 µg/mL per disk did not show any inhibitory effect against *Candida* species in the control agar plates without fluconazole (Figure 13A,E,H) and in the presence of fluconazole (Figure 13B,C,F,G,I,J). These results indicated the lack of synergistic fungicidal activity of BCA and fluconazole.

## 3. Discussion

### 3.1. BCA Inhibits the Growth of Candida Species

Natural products from plants have often been reported to have potent antifungal properties in recent years [53,54,55]. Flavonoids are a subdivision of polyphenols, a versatile class of natural compounds that represent secondary metabolites from higher plants and are abundant in human diet [39]. Flavonoids are generally considered as low-toxic phytochemicals and their various biological effects, including antifungal activity, have been reported [39]. The BCA isoflavone has been intensively studied due to its therapeutic potential against various infectious diseases, including bacterial and parasitic infections [1]. The antifungal activity of BCA has been proven against plant pathogens, including *Penicillum italicum*, *Rhisoctonia solani*, and *Sclerotium rolfsii* [19,20]. Extracts from roots of *Virola surinamensis* containing BCA displayed antifungal activity against *Cladosporium cladosporioides* [21]. Moreover, BCA has been shown to be active against human pathogen *Candida albicans* [22].

Of the 300 fungal species known to cause disease in humans, 20 species are particularly frequent, including *Candida* spp. [28,29]. *C. albicans* is the most commonly isolated *Candida* in clinical cases of invasive fungal infections. However, in the last two decades, the number of infections caused by non-*C. albicans Candida* species has increased significantly [49,56,57]. The pathogenic mechanisms of non-*C. albicans Candida* are not as well understood as those of *C. albicans*, where more extensive research has been carried out [58]. Adhesion, biofilm, secretion of enzymes, immune evasion strategies, and resistance to antifungals play important roles in their pathogenicity [49]. Here, we demonstrated that BCA was effective against planktonic cells of six *Candida* species, including *C. albicans*, *C. parapsilosis*, *C. glabrata*, *C. tropicalis*, *C. auris*, and *C. krusei*, with MIC_50_ values ranging from 125 µg/mL to 500 µg/mL and with MIC_90_ ranging from 250 µg/mL to 1000 µg/mL. These results are comparable to a previous study in which the MIC value for *C. albicans* was 1690 µg/mL [22]. However, BCA had weak antifungal activity against different *Candida* species compared to other isoflavones. Some isoflavones have been known for their strong antifungal activity, e.g., sedonan A, which inhibited *C. albicans* and *C. glabrata* at MIC values of 15 µg/mL and 7.6 µg/mL, respectively, and dorsmanin extracted from *Dorstenia mannii* has been found to have antifungal activity against *C. albicans* with an MIC of 64 µg/mL [39,59,60]. On the other hand, we proved in this study that BCA significantly inhibited biofilm formation and had a destructive effect on the mature biofilms formed by all the tested *Candida* species.

### 3.2. BCA Inhibits the Biofilm of Candida Species

The first stage of the formation of *Candida* biofilm is early cell adhesion. Our results showed that BCA prevented cell adhesion. The biofilms formed in this phase by all the tested *Candida* species were significantly reduced by the BCA treatment at concentrations of 125–1000 µg/mL compared to the control biofilms, as evidenced by the MTT assays and crystal violet staining. We further explored the effect of BCA on the hydrophobic properties of six *Candida* species. The cell surface hydrophobicity (CSH) of biofilm-forming *Candida* species is important for colonization, adhesion, biofilm growth, and antibiotic resistance. Greater CSH mostly results in higher levels of these properties, i.e., important elements of pathogenesis [61]. BCA was found to reduce the CSH of the *Candida* species, which undoubtedly prevents adhesion and is a good strategy to block the development of fungal infection.

The effects of BCA were also investigated in the initial stage of biofilm formation and in the mature biofilm in the *Candida* species. Biofilms formed by *Candida* are even a thousand times more resistant to most of the commonly used antifungal drugs than planktonic cells. Unfortunately, higher concentrations of antifungals are not desirable due to their toxicity-related side effects [62]. Our results of two tests, i.e., the MTT assay and CV staining, showed that BCA inhibited the formation of biofilms and destroyed the mature biofilms at the same concentrations that were effective against planktonic cells of all the tested *Candida* strains (125–1000 µg/mL).

Moreover, we noted that BCA used at the sub-inhibitory concentrations (125 µg/mL and 250 µg/mL) also contributed to the reduction of hyphae in *Candida* species forming hyphae (*C. albicans*, *C. parapsilosis*, *C. krusei*, *C. tropicalis*, and *C. auris*) in the four hyphal-inducing media. Hyphae are important structures of *Candida* spp. biofilms and are essential for successful fungal colonization and invasion of the host, as the greater the ability of a fungus to adhere to the host, produce hyphae, and form biofilms, the higher the chances of persistent and severe infections [63,64,65].

To gain insight into the molecular mechanism of the BCA-mediated hyphal growth inhibition and biofilm formation inhibition, the expression profile of hypha-specific and biofilm-related genes in *C. albicans* cells were analyzed. Among the tested genes were adherence-related genes *ALS1* (agglutinin-like sequence 1) and *ALS3* (agglutinin-like sequence 3), as well as hyphae-related genes *HWP1* (hyphal wall protein 1), *HYR1* (hyphally regulated gene), *SAP4* (secreted aspartyl proteinase 4), *CPH* (transcription factor), and *ECE1* encoding candidalysin, which has been linked to such *C. albicans* virulence factors as adhesion, biofilm formation, and filamentation properties. Moreover, we examined the expression level of regulatory genes, including *EFG1*, a transcription factor in the Ras1–cAMP–Efg1 pathway positively regulating the expression of hypha-specific genes, and *BCR1*, a biofilm master regulator [66]. Our results showed that BCA down-regulated the expression levels of all these genes and inhibited adherence, hyphal growth, and biofilm formation also through this pathway of action.

These results are very important, because the majority of *Candida* infections are associated with the ability of the yeast to form biofilms. The biofilm-forming capability of *Candida* spp. contributes significantly to their resistance against the currently available antifungal drugs, making it difficult to eradicate the infection [67]. It is known that natural products have a very strong prospect to deliver effective chemotherapeutics for the treatment for biofilm-involving candidiasis [68]. Hence, the identification of a new active product from plants, BCA, with inhibitory potential against biofilm formed by pathogenic *Candida* species is extremely promising for the effective treatment of candidiasis.

### 3.3. BCA Causes Physical Destruction of Cell Membranes and Cell Walls

Our research indicated that BCA acts on the *C. albicans* cell wall. The structural integrity of the yeast cell wall is necessary for the survival and reproduction of cells. A damaged cell wall causes osmotic disorders in the fungal cell, rupture of the cell membrane, outflow of cytoplasmic content and, consequently, cell death. Glucan together with chitin are the main structural components of the fungal cell wall responsible for the integrity and physical strength of this structure. These polysaccharides are present in fungi, but not in human cells, making the fungal cell wall an interesting target for selective and safe antifungal therapies [69,70]. Our data suggest that the possible mechanism of action of BCA involves its activity towards (1,3)-*β*-D-glucans and chitin in *C. albicans* cells, as demonstrated by the aniline blue and calcofluor white staining, respectively. The consequence of cell wall disruption is damage to the cell membrane and disturbances in its proper functioning. Many cellular processes involved in cell growth and function are associated with changes in membrane characteristics. As presented in this study, BCA causes deformation of the plasma membrane structure in *C. albicans* cells. The modification of the biophysical properties of the cell membrane is caused by changes in the lipid composition [71]. We used DPH to determine whether BCA influenced the lipid arrangement in the *C. albicans* cell membrane. DPH has the ability to bind to the membrane. It is often used in measurements of fluorescence anisotropy and provides insight into membrane fluidity and lipid ordering [72]. The results of the treatment of *Candida* cells with BCA showed a significant reduction in the fluorescence intensity caused by membrane deformation. To check whether BCA also affected the permeability of the *C. albicans* cell membrane, a propidium iodide (PI) assay was performed. PI penetrates the damaged fungal cell membrane and binds to DNA [73]. As shown by our results, there was an increase in PI in the BCA-treated *Candida* cells. These results suggest that BCA is involved in changes in membrane permeability through alteration of the composition and disruption of the physical integrity of *C. albicans* cell membrane. In this respect, another isoflavone, glabridin isolated from *Glycyrrhiza glabra*, showed similarities in its action, as it affected cell membrane permeability, which resulted in cell envelope damage [74].

### 3.4. BCA Does Not Interact with Fluconazole

Finally, the possible synergistic antifungal effect of BCA and fluconazole against *C. albicans*, *C. glabrata*, and *C. auris* was investigated in this study. This synergistic drug combination has been proved to be a valid and pragmatic strategy to seek drugs with a novel mode of action. It can potentially reduce the dose of single drug usage with increased drug-efficacy and, subsequently, diminish drug toxicity. Furthermore, the development of drug resistance can be slowed down by the two- or multi-target strategy [75]. The agar disk diffusion assays did not indicate a synergistic effect of BCA and fluconazole against fluconazole-resistant *C. albicans*, *C. glabrata*, and *C. auris*, although previous research showed that other isoflavones had a synergistic effect with antifungal drugs. Glabridin and nystatin have been shown to be active against oral *C. albicans* [76]. Moreover, glabridin was found to have a synergistic effect with fluconazole [74].

## 4. Materials and Methods

### 4.1. Strains

Six *Candida* species from the American Type Culture Collection (*Candida albicans* ATCC 10231, *Candida glabrata* ATCC 15126, *Candida parapsilosis* ATCC 2099, *Candida krusei* ATCC 14243, *Candida tropicalis* ATCC 13803, and *Candida auris* ATCC MYA-5001) were used to test the antifungal activities of biochanin A. All strains were grown on YPD agar (1% yeast extract, 1% peptone, 2% dextrose, 2% agar) and before each assay a colony was transferred into YPD medium for propagation at 37 °C overnight with a rotation of 200 rpm.

### 4.2. Chemicals and Microbiological Media

MTT (3-(4, 5-dimethylthiazol-2yl)-2, 5-diphenyl-2H-tetrazoliumbromide), menadione (2-methyl-1, 4-naphthoquinone), biochanin A (purity ≥ 95.0%) (dissolved in DMSO at a concentration not exceeding 1% in all assays), resazurin, fetal bovine serum (FBS), and other reagents were purchased from Sigma-Aldrich (St. Louis, MO, USA). Spider medium (1% nutrient broth, 1% mannitol, 0.2% K_2_PO_4_, 1% fetal bovine serum, pH 7.2), Sabouraud Dextrose (SD, Biocorp, Warsaw, Poland), and RPMI 1640 medium (with L-glutamine, without sodium bicarbonate (Sigma-Aldrich, St. Louis, MO, USA) were used as well. RPMI-1640 medium was buffered with 0.165 M MOPS (3-(N-morpholino)propanesulfonic acid) to pH 7.0.

### 4.3. Antifungal Susceptibility Assay

Assays for antifungal susceptibility testing were performed in RPMI 1640 medium. Antifungal susceptibility testing was performed according to the Clinical and Laboratory Standards Institute (CLSI) guidelines for broth microdilution CLSI-M27-A3 [77] and described by Khabnadideh et al. [78]. Biochanin A (BCA) was used in a concentration range from 1 µg/mL to 1000 µg/mL. In order to eliminate the error in the assessment of antifungal activity, the medium was supplemented with a 10% resazurin solution. The plates were then incubated at 30 °C for 4 h and the inhibition of fungal growth was determined by measuring fluorescence (excitation, 544 nm; emission, 590 nm) [79]. MIC (minimal inhibitory concentration) endpoints were determined to be 50% (MIC_50_) and 90% (MIC_90_) growth reduction.

### 4.4. Adhesion Assay

Overnight grown cultures of *Candida* spp. in YPD medium were concentrated and diluted to 1 × 10^6^ cells/mL in RPMI 1640 medium. One hundred milliliters of such a cell suspension was placed in a 96-well plate and different volumes of a BCA solution were added into each well to reach the final concentrations of 125 µg/mL, 250 µg/mL, 500 µg/mL, and 1000 µg/mL. After 2 h of incubation at 37 °C, each well was washed with PBS (phosphate buffered saline, pH 7.4) three times. The biomass of adherent cells on the bottom of each well was stained with crystal violet and the viability of adherent cells in each well was determined by MTT reduction assay.

### 4.5. Crystal Violet Assay

Staining of adherent cells with 0.5% (*w*/*v*) crystal violet (CV) was carried out for 20 min at room temperature. A bench rocker with a frequency of 20 oscillations per minute was used. The plate was rinsed thoroughly with tap water to remove excess dye. Uncovered plates were air-dried at room temperature for 24 h. Subsequently, 200 mL of methanol were added to each well and the plates were covered with lids and incubated on a bench rocker for 20 min at room temperature. Crystal violet dye bound to the biofilms was quantified via measurement of the absorbance of the solution at 570 nm using a microplate reader (BioTek Synergy H1 Microplate Reader, Winooski, VT, USA). The biomass inhibition percentage was calculated based on the values obtained in the experimental and control groups.

### 4.6. MTT Assay

The adherent *Candida* cells in each well were treated with a mixture containing 40 µL of MTT (1 mg/mL), 2 µL of 0.4 mM menadione, and 158 µL of PBS in order to determine their viability. After incubation at 37 °C for 3 h, absorbance at 570 nm was measured using a microplate reader (BioTek Synergy H1). The percentage of viability inhibition was calculated based on the values of the experimental group and the growth control.

### 4.7. Cell Surface Hydrophobicity (CSH)

The cell surface hydrophobicity of the *Candida* cells was determined using the water-hydrocarbon two-phase assay. Cell suspensions (2 mL, 1 × 10^6^ CFU/mL) supplemented with 125 µg/mL, 250 µg/mL, 500 µg/mL, and 1000 µg/mL of BCA were incubated in glass tubes for 24 h at 37 °C. Next, the suspensions were centrifuged for 10 min at 3000× *g*, and the fungal cells were resuspended with sterile PBS at OD_600_ of 1.0. A 1.2 mL aliquot of the strain solution was transferred into a clean glass tube and 0.3 mL of octane was added (Sigma Aldrich, St. Louis, MO, USA). The solutions were vortexed for 3 min and separated into two distinct phases. The OD_600_ of the aqueous phase was determined. The control was set as the OD_600_ of the aqueous phase without the octane overlay. The relative hydrophobicity was expressed as the percentage change in the optical density (OD_600_) of the BCA-untreated cells (100%) [80].

### 4.8. Morphological Transition

To explore the effects of BCA on the hyphal growth of *Candida* species, overnight-grown fungal cultures were centrifuged and then seeded into various hyphal-inducing media (RPMI 1640 medium, Spider medium, GlcNAc medium, and SD medium with 10% FBS) at the concentration of 10^6^ cells/mL. The yeasts were incubated with BCA at concentrations of 125 µg/mL and 250 µg/mL at 37 °C for 4 h. Cultures without BCA were the controls. The cellular morphologies of *Candida* species were inspected under a light inverted microscope and imaged using a digital camera.

### 4.9. Inhibition of Biofilm Formation

The *Candida* cells were cultured as biofilms in polystyrene flat-bottomed microtiter plates. Cell suspensions prepared in RPMI 1640 medium at a cell density of 2 × 10^6^ cells/mL were transferred into microtiter plates wells (100 mL per well) [62]. The effect of BCA on the ability to form biofilms was assessed in the presence of 100 mL of the compound used at different concentrations (125–1000 µg/mL). In total, 100 mL of RPMI 1640 medium containing 1% DMSO but supplemented with the tested compound served as a control. After 24 h incubation at 37 °C, the medium was aspirated from the wells and the biofilms were washed three times with PBS to remove planktonic-phase cells. The biofilms were stained with MTT and crystal violet according to the methods described above.

### 4.10. Mature Biofilm Eradication

*Candida* biofilm cell suspensions were prepared in RPMI 1640 at a density of 1 × 10^6^ cells/mL. Next, 100 µL of the suspensions were transferred into the wells and incubated at 37 °C for 24 h. Afterwards, non-adherent cells were gently removed and the wells were rinsed three times with PBS and filled with 100 µL of two-fold dilutions of BCA in RPMI 1640 (125–1000 µg/mL). The negative control was prepared by adding 100 µL of RPMI 1640 medium containing 1% DMSO into some of the biofilm-containing wells. Next, the microtiter plates were incubated at 37 °C for 24 h. In the next step, the medium was collected from the wells and the biofilms were washed as described previously to remove nonadherent cells. The crystal violet staining of dry biofilms and the MTT assay were performed according to the methods cited above.

### 4.11. Relative Quantification by Real-Time Reverse Transcriptase qRT-PCR

The expression of *ALS1*, *ALS3*, *BCR1*, *CPH1*, *ECE1*, *EFG1*, *HWP1*, *HYR1*, and *SAP4* genes was assessed using the real-time PCR technique following the BCA treatment. The *C. albicans* cells were treated with 125 µg/mL and 250 µg/mL of BCA or 1% DMSO (control) during propagation in liquid culture in Spider medium supplemented with 10% fetal bovine serum (FBS). After 4 h incubation at 37 °C, the cells were collected and total RNA was isolated using the YeaStar RNA kit (Zymo Research, Irvine, CA, USA) in accordance with the manufacturer’s instructions. Next, cDNA was synthesized with the use of a Smart First Strand cDNA Synthesis Kit (EurX, Gdańsk, Poland) as specified in the manufacturer’s instructions. TaqMan gene expression assays (Lot: 170255, designed by the manufacturer, ThermoFisher Scientific, Swindon, UK) and the Fast Probe qPCR Master Mix (EurX, Poland) were used to PCR detection of transcripts. The cDNA samples were first pre-treated with uracil-N-glycosylase at 37 °C for 2 min to degrade any dUMP-containing PCR products. Next, they were subjected to the initial denaturation process at 95 °C for 3 min, 40 amplification cycles with denaturation at 95 °C for 10 s, and annealing/extension at 60 °C for 30 s with the use of QuantStudio3 (Applied Biosystems, San Francisco, CA, USA). The 2-(∆∆Ct) method was used for calculation of the relative level of expression of the analyzed genes using *ACT1* as a reference gene [80].

### 4.12. Aniline Blue and Calcofluor White Staining

The aniline blue and calcofluor white (disodium salt of 4,4′-bis-[4anilino-bis-diethyl-amino-S-tri-azin-2-ylamino]-2,2′stilbene-disulfonic acid) staining methods were used to visualize the effect of BCA on the cell wall of *C. albicans*. Aniline blue has the ability to bind to (1,3)-*β*-D-glucans, whereas calcofluor white binds to chitin in the *C. albicans* cell walls. The yeast cells at the exponential phase were harvested by centrifugation at 4500× *g* at 4 °C for 5 min. Next, the cells were washed twice and resuspended in PBS. BCA at concentrations of 125 µg/mL and 250 µg/mL and 1% DMSO as a control were added to the cell suspensions and incubated at 37 °C for 4 h. The cells were harvested and washed in PBS. Next, the cell density in each experimental group was adjusted to 1 × 10^8^ cells/mL and the cells were resuspended in an aniline blue solution (0.1%) or calcofluor white (0.1%). The samples were stained for 30 min, washed with PBS, and fluorescence was measured in a black 96-well microplate using a spectrofluorometer at 370 nm excitation and 509 nm emission wavelengths for aniline blue and at 370 nm excitation and 440 nm emission wavelengths for calcofluor white [62,81,82,83].

### 4.13. PI Staining

The effect of BCA on the cell membrane of *C. albicans* was tested using PI (propidium iodide) staining. In brief, 1 × 10^7^ cells/mL in SD medium obtained from overnight grown cultures were treated with different concentrations of BCA (125 µg/mL and 250 µg/mL) at 37 °C for 3 h. The volume of DMSO used for incubation of the control cells was the same as that of BCA. Then, the cells were washed with PBS (pH 7.4) and all the suspensions were adjusted to an OD at 600 nm for 1 × 10^8^ cells/mL. To assess the effect of BCA on the membrane integrity in *C. albicans*, the yeast cells were incubated with the fluorescence probe PI (final concentration: 10 µM) at room temperature for 10 min in the dark. Fluorescence was measured at 488 nm excitation and 617 nm emission wavelengths using a spectrofluorometer (BioTek Synergy H1) [52].

### 4.14. DPH Assay

The effect of BCA on the structure of the yeast cell membrane was investigated by analysis of binding of 1,6-diphenyl-1,3,5-hexatriene (DPH) to the membrane. *C. albicans* cells (1 × 10^7^ cells/mL) were incubated in the presence of 125 µg/mL and 250 µg/mL BCA at 37 °C on a shaking incubator at 200 rpm for 3 h. The same volume of DMSO as in the BCA-supplemented variant was used for incubation of the control cells. The cells were collected from the growth medium by centrifugation at 4000× *g* for 5 min. Next, they were washed and resuspended in PBS (pH 7.4). Each suspension was adjusted to an optical density at 600 nm for 1 × 10^8^ cells/mL. To assess the effect of BCA on 1,6-diphenyl1,3,5 hexatriene (DPH) binding to the *C. albicans* membrane, the cells were incubated with the fluorescence probe DPH (final concentration: 2 µM) at room temperature for 30 min in the dark. Afterwards, the samples were washed with PBS (pH 7.4), and fluorescence was measured in a black 96-well microplate at 360 nm excitation and 460 nm emission wavelengths with the use of a spectrofluorometer (BioTek Synergy H1) [52].

### 4.15. Agar Disk Diffusion Test

The interactions of BCA and fluconazole against three fluconazole-resistant species, i.e., *C. albicans*, *C. glabrata*, and *C. auris*, were tested using the agar diffusion test. The aliquot suspension of the cells (10^6^ cells/mL) was spread uniformly onto a yeast peptone dextrose (YPD) agar plate with or without BCA (125 µg/mL and 250 µg/mL) or with or without fluconazole (8 µg/mL and 32 µg/mL). Then, 6-mm paper disks impregnated with fluconazole at concentrations of 8 µg/mL, 16 µg/mL, 32 µg/mL, and 64 µg/mL were placed on agars with a BCA-coated surface. In the same way, paper disks soaked with BCA at 125 µg/mL, 250 µg/mL, and 500 µg/mL were prepared and placed on agars with fluconazole. A volume of 5 µL DMSO was used in the control disks. Photographs were taken after incubation at 37 °C for 48 h [84].

### 4.16. Statistical Analysis

All data are expressed as a mean ± SD (standard deviation) of three independent experiments. Statistical significance between the treated and control groups were analyzed by Student’s *t*-test using GraphPad Software version 9.1.1. (San Diego, CA, USA). The *p* value < 0.05 was considered statistically significant.

## 5. Conclusions

This was the first study showing the antifungal activity of BCA against six *Candida* species, including *C. albicans*, *C. parapsilosis*, *C. glabrata*, *C. tropicalis*, *C. auris*, and *C. krusei*. BCA also inhibited adhesion, biofilm formation, and morphological transition, decreased the CSH, destroyed mature biofilm, and reduced the expression level of genes associated with hyphal and biofilm formation. Furthermore, the BCA treatment caused physical destruction of the *C. albicans* cell membrane and cell wall. These properties predispose BCA for assessment of its potential as an antifungal compound.

## Figures and Tables

**Table 1:** Biochanin A minimum inhibitory concentrations (MIC_50_ and MIC_90_).

*Candida* Species	MIC_50_ (µg/mL)	MIC_90_ (µg/mL)
*C. albicans*	250	500
*C. glabrata*	500	1000
*C. parapsilosis*	250	500
*C. krusei*	125	250
*C. tropicalis*	250	500
*C. auris*	250	500

## Data Availability

Data are contained within the article.
